# Supplementary material for: Effectiveness and Safety of Ayurvedic Medicines in Type 2 Diabetes Mellitus Management: A Systematic Review and Meta-Analysis
Source: Front Pharmacol. 2022 Jun 8;13:821810. doi: 10.3389/fphar.2022.821810 (PMC9213670; doi:10.3389/fphar.2022.821810)
Supplement: Supplementary file 1 [file DataSheet1.zip › Supplementary Material/Forest and Funnel Plots/Trigonella foenum-graecum (versus OAD)/HDL-C.pdf]

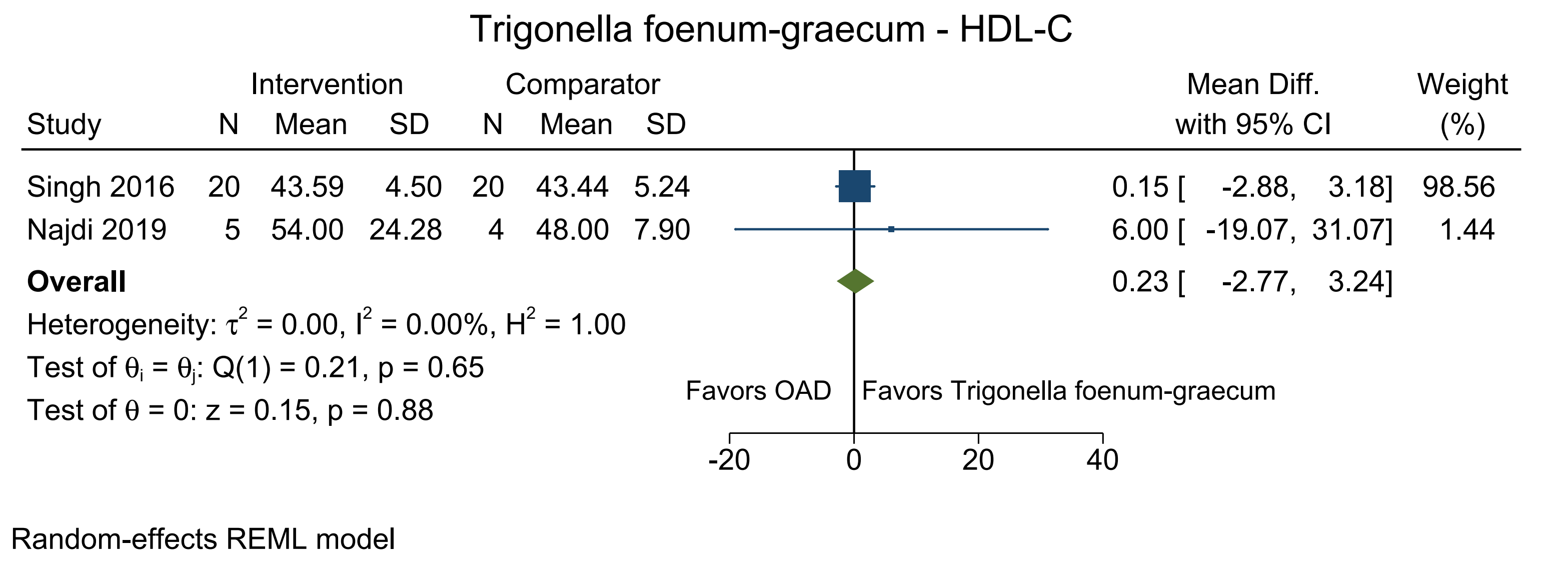
Trigonella foenum-graecum - HDL-C
Intervention
Comparator
Mean Diff.
with 95% CI
Weight
(%)
Study
N
Mean
SD
N
Mean
SD
Singh 2016 20 43.59 4.50 20 43.44 5.24
0.15 [-2.88, 3.18] 98.56
Najdi 2019 5 54.00 24.28 4 48.00 7.90
6.00 [-19.07, 31.07] 1.44
Overall
0.23 [-2.77, 3.24]
Heterogeneity: τ² = 0.00, I² = 0.00%, H² = 1.00
Test of θi = θj: Q(1) = 0.21, p = 0.65
Test of θ = 0: z = 0.15, p = 0.88
Favors OAD
Favors Trigonella foenum-graecum
-20
0
20
40
Random-effects REML model
